# Ecological strategies of biological and chemical control agents on wildfire disease of tobacco (*Nicotiana tabacum* L.)

**DOI:** 10.1186/s12866-021-02237-8

**Published:** 2021-06-17

**Authors:** Tianbo Liu, Yabing Gu, Zhicheng Zhou, Zhenghua Liu, Huaqun Yin, Chong Qin, Tuyong Yi, Jiemeng Tao

**Affiliations:** 1 College of Plant Protection, Hunan Agricultural University, Changsha, China; 2 Central South Agricultural Experiment Station of China Tobacco, Changsha, China; 3 School of Minerals Processing and Bioengineering, Central South University, Changsha, China; 4 China Tobacco Gene Research Center, Zhengzhou Tobacco Research Institute of CNTC, Zhengzhou, China

**Keywords:** Biological control agent, Endophytic bacterial community, Community assembly, Ecological process

## Abstract

**Background:**

To investigate the ecological effects of chemical and biological control methods on tobacco wildfire disease, a plot field experiment was conducted to compare the control efficiency and mechanisms of a chemical pesticide (kasugamycin wettable powder, KWP) and a biological control agent (BCA) through high-throughput sequencing of bacterial 16S rRNA genes.

**Results:**

The results showed that the BCA displayed better performance in decreasing the disease index and morbidity of tobacco than the chemical pesticide. By monitoring the endophytic community within tobacco leaves, it was found that the control effects of these two methods might be mediated by different changes in the endophytic bacterial communities and community assembly patterns. The application of either method decreased the taxonomic diversity of the leaf endophytic community. Compared to the BCA, KWP showed a more significant effect on the endophytic community structure, while the endophytic community treated with the BCA was able to return to the original state, which presented much lower disease infection. The disease control efficiency of KWP and BCA treatments might be achieved by increasing the abundance of *Sphingomonas* and *Streptophyta*, respectively. Furthermore, an analysis of the ecological processes in community assembly indicated that the BCA strengthened the homogeneous and variable selection, while KWP enhanced ecological drift.

**Conclusions:**

The results suggested different control mechanisms between KWP and BCA treatments, which will help in developing diverse ecological strategies for plant disease control.

**Supplementary Information:**

The online version contains supplementary material available at 10.1186/s12866-021-02237-8.

## Background

Wildfire disease caused by *Pseudomonas syringae* pv. *tabaci* is a typical, destructive leaf disease of tobacco [1]; hence, a great amount of effort has been made to control it, including spraying chemical pesticides, selecting wildfire-resistant cultivars, and using microbial agents [2]. The use of chemical pesticides has led to the emergence of environmental pollution and resistant bacterial strains [3], biological control of plant pathogens has been viewed as an ecologically mild and environmentally safe alternative. To date, a large number of biological control agents (BCAs), such as *Bacillus* spp., *Pseudomonas* spp., and *Trichoderma* spp., have been commercialized for various crops [4, 5].

Many studies have reported that biological and chemical methods have different control efficiencies against plant disease [6, 7], but the mechanisms of different control methods for the microbial community are rarely reported. Previous studies indicated the disease resistance mechanisms of chemical agents, including the reduction of membrane permeability, inhibition of extracellular protease secretion, induction of gene expression, phytoalexin production and induction of systemic resistance, could be considered a direct antimicrobial effects [8, 9]. The disease suppression function of BCAs occurs via reciprocal inhibition relationships between biological species [10]. The phyllosphere is considered one of the most extensive ecosystems colonized by microorganisms [11], and interactions among the microbial community, host plant, and exogenous agents are inseparable. Thus, an acute understanding of the mechanisms underlying these interactions is necessary, regardless of the method that is utilized.

Compared to studies on soil microbiota, the few studies of phyllosphere microbiota conducted to date have primarily focused on fruit and vegetable crops [12, 13]. Our previous study showed that spraying BCAs on plant leaves greatly altered the phyllosphere microbial community and suppressed the outbreak of tobacco wildfire disease [14]. We speculate that the control mechanism of BCAs might be attributed to their effects on phyllosphere microorganisms, both epiphytic and endophytic. Compared with epiphytic microbes, endophytic microbes, which occupy niches within the plant root, stem, and leaf tissues, have higher compatibility with plants, and may greatly influence plant growth and health [15, 16]. Recently, a biological control treatment by *Methylobacterium* sp. showed disease prevalence in three cultivars of potato (*Solanum tuberosum L*.) was associated with shifts in the endophytic microbial community [17]. Thus, alteration of the endophytic community might be important in disease suppression.

Reports have suggested that microbial communities are driven by different ecological assembly processes [18, 19]. Vellend [20] proposed the following four fundamental ecological processes to explain patterns in microbial community assembly: including selection, dispersal, speciation and ecological drift [21]. To quantify the relative contributions of these processes in microbial community assembly, Stegen et al. [22, 23] reported a null-modeling-based statistical framework, which has been applied to a number of microbial communities [24–26]. For instance, by detecting the community assembly processes of attached and waterborne microbiomes across inland hyporheic, nearshore hyporheic and river regions, Graham et al. [27] showed that river microbiomes were assembled via homogeneous selection while more than 90% of the dissimilarity in microbiomes between nearshore and inland zones was caused by variable selection. Various deterministic factors, such as changes in environmental conditions [28], habitat conditions in hosts [29], and species traits and microbial interactions [30], are important in governing microbial community structure. Although alteration of microbial communities by applying exogenous chemical or biological agents has been studied recently, the assembly processes governing the phyllosphere microbiome in this case remain unclear.

Our research had four primary objectives: (a) identify the control efficiency of tobacco wildfire disease by both chemical and biological agents; (b) determine whether chemical and biological control agents would alter the structure of the phyllosphere endophytic microbial communities; (c) determine whether there are specific groups of organisms present relative abundances that are correlated to disease incidence; and (d) identify the assembly processes that generate these community patterns over time. We hypothesize that (a) the agent type (i.e., chemical or biological) generates differences in disease suppression; (b) the structure of the phyllosphere endophytic microbial communities is altered in distinct ways by applying different agents; (c) several endophytic microorganisms associated with tobacco wildfire disease are activated; and (d) exogenous agents facilitate microbial selection.

## Results

### Tobacco disease incidence estimating

In the present study, morbidity and disease indices were used to describe wildfire infection of tobacco plants. The indices showed similar trends between the two treatments as compared to the control (Tables S1 and 1). On Day 21, compared to CK, the treatments displayed significantly (LSD test, *P* < 0.05) decreased morbidity and disease index values, but the morbidity and disease indices of the BCA were further lower than that of the chemical control agent (kasugamycin wettable powder, KWP) (LSD test, *P* < 0.05). The control efficiency of the BCA reached 67.99% based on the disease index, which was significantly higher than that of KWP (LSD test, *P* < 0.05). These results indicated that both biological and chemical agents played positive roles in inhibiting wildfire disease in tobacco, and that the BCA was more effective than KWP (LSD test, *P* < 0.05).

**Table 1:** Tobacco wildfire disease index under different treatments

Treatment	0 day	7 day	21 day
BCA	3.37 ± 0.27a	3.74 ± 0.42b	4.59 ± 0.55c
KWP	3.22 ± 0.24a	3.79 ± 0.71b	7.08 ± 1.15b
CK	3.29 ± 0.53a	4.24 ± 1.07a	14.34 ± 0.59a

Different letters indicate significant difference at 0.05 by multiple comparisons based on the means of least significant difference and a grouping of treatments

### Responses of endophytic microbial communities to the BCA and KWP

**Fig. 1:**
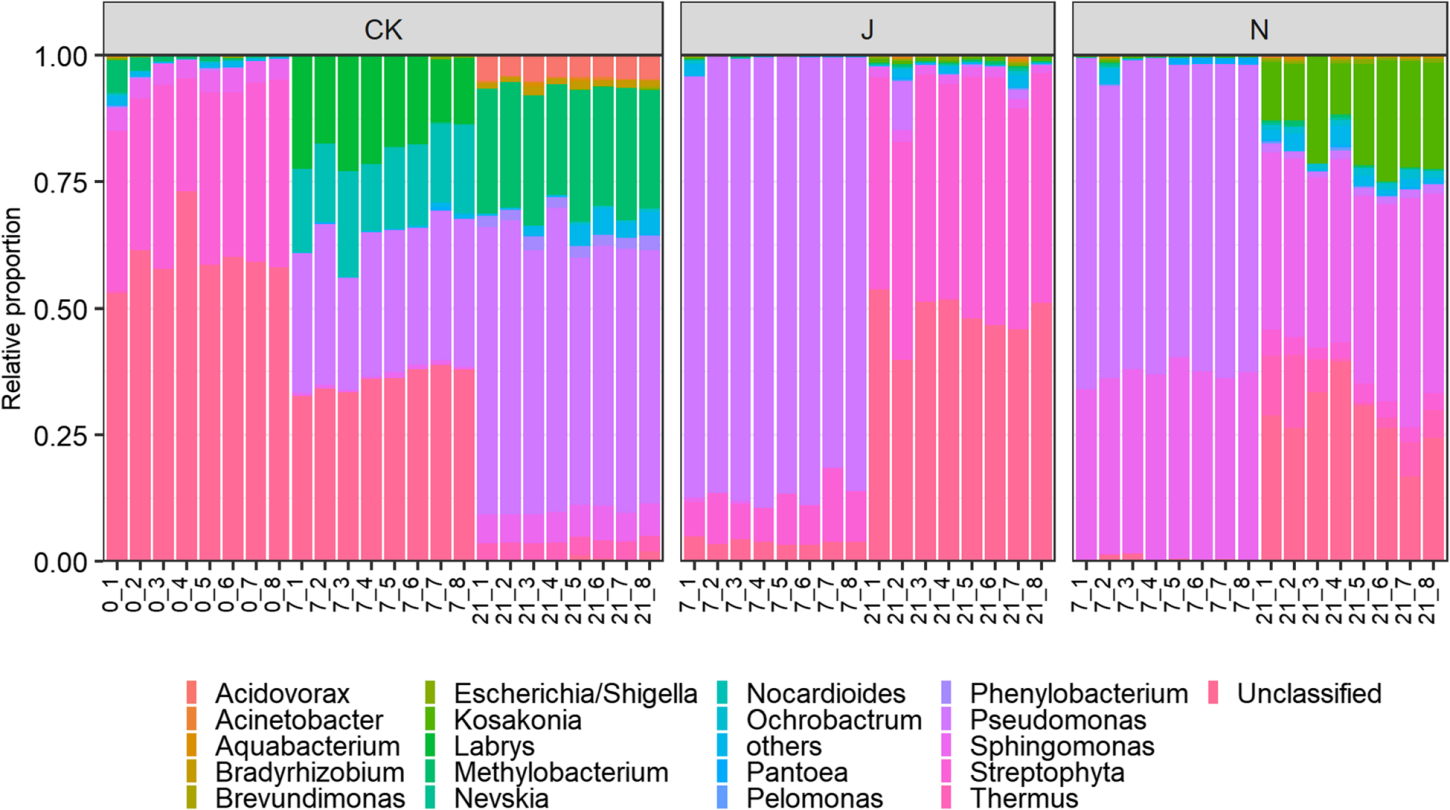
Average relative abundance of bacterial genera across different treatments, including control group (CK) and treatments of biological control agent (BCA) and chemical control agent (KWP). The number in front of “_” is the number of experimental days (0 day for CK group labeled as “Original”), while the number behind “_” is the serial number of the replicates

In the present study, a total of 1,019,145 high quality sequences were obtained. To avoid any effects caused by sequencing depth, all samples were rarefied randomly to 20,000 sequences (Figure S1). The sequencing data results showed that 625 OTUs occurred across all samples, which included 256 genera. In the endophytic microbial communities, the genera *Streptophyta, Sphingomonas, Pseudomonas, Methylobacterium, Ochrobactrum, Tatumella, Labrys, Acidovorax, Kosakonia, Bradyrhizobium, Nocardioides* and *Phenylobacterium,* had an average relative abundance of more than 1% (Fig. 1). However, the dominant genera were quite different among treatments with tobacco growth. For example, the most abundant genus in the original (pretreatment tobacco) samples was *Streptophyta* (32.40%), whereas *Pseudomonas* was the dominant genus in CK_7 (27.94%), CK_21 (53.67%), BCA_7 (85.91%), and KWP_7 (60.97%). By Day 21, *Streptophyta* became the dominant genus again in the BCA treatment (44.67%), and the dominant genus shifted to *Sphingomonas* in the KWP treatment (37.61%). *Sphingomonas* also accounted for a high proportion of KWP (36.37%) on Day 7. In addition, changes of the relative abundance of *Pseudomonas,* which included the wildfire disease pathogen, were quite different among treatments (Figure S2). PERMANOVA revealed that time (F = 553.6, *P* < 2e^− 16^), and treatment (F = 2686.2, *P* < 2^e-16^) both contributed significantly to the change in *Pseudomonas*. The relative abundance of *Pseudomonas* increased continuously and significantly in the CK with the growth of tobacco (LSD test, *P* < 0.05). However, in the BCA and KWP treatments, it increased significantly by Day 7 and then clearly decreased by Day 21 (LSD test, *P* < 0.05).

**Fig. 2:**
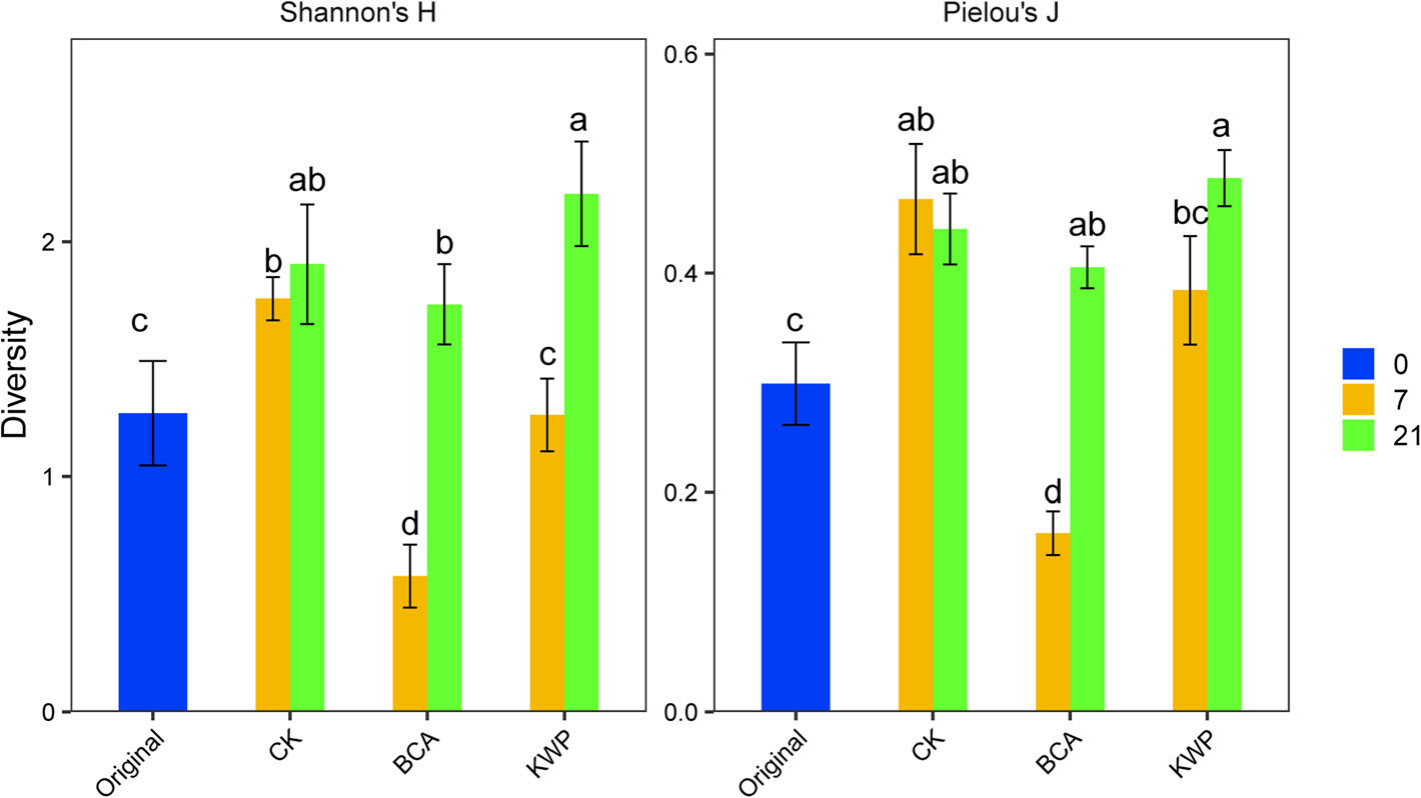
Alpha diversity of microbial communities based on OTU level across the control groups (CK), biological control agent (BCA) and chemical control agent (KWP) groups. Diversity indexes of Shannon-Weiner’s index (*H*) and Pielou evenness (*J*) are shown. Results are means and S.D. of eight replicates. Different letters above the column indicated the differences among samples are significant at the level of 0.05

The α-diversity indices, including Shannon-Weiner’s index (*H*) and Pielou evenness (*J*), were used to evaluate the changes in microbial community diversity. The indices showed that there were differences between treatments and over time (Fig. 2). Across all treatments, the α-diversity indices increased and reached their highest value on Day 21, whereas the process of change showed a different trend. The α-diversity indices increased continuously with time in the CK, while the indices in the KWP treatment did not show significant change (LSD test, *P* < 0.05) on Day 7 compared to the original samples (Day 0), but had increased by Day 21. Indices of BCA decreased significantly on Day 7, but increased rapidly by Day 21 (LSD test, *P* < 0.05). It is worth noting that the Shannon-Weiner index of the BCA was significantly (LSD test, *P* < 0.05) lower than that of the CK on both Day 7 and Day 21, and Pielou evenness of the BCA treatment was significantly lower than CK on Day 7.

**Fig. 3:**
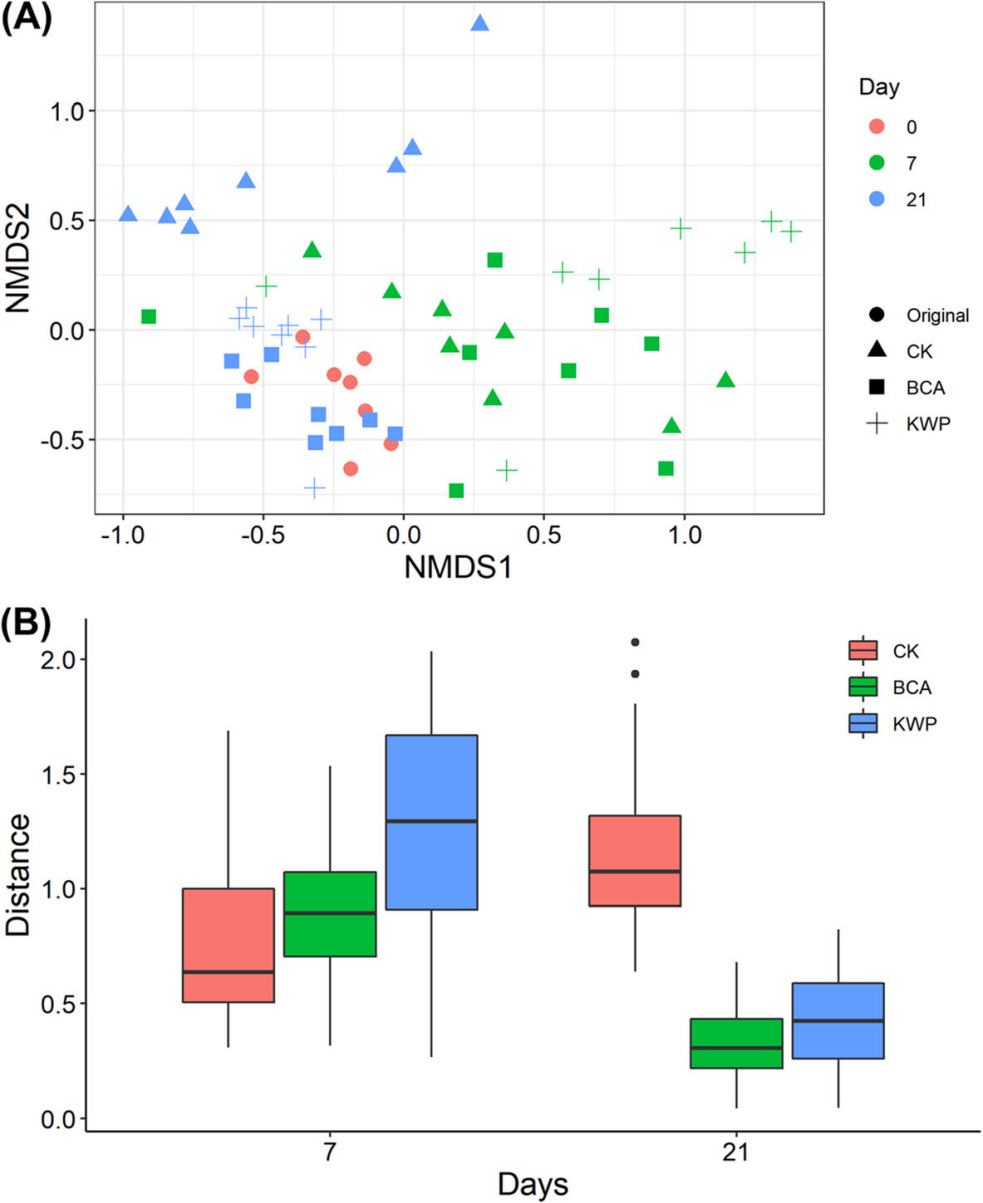
Non-metric multidimensional scaling (NMDS) plots (**A**) and the boxplot of within-group distance based on NMDS plots (**B**)

For the community structure of endophytic microbes, time might be the significant influencing factor based on ADONIS (*R*^2^ = 0.0942, *p* = 0.007) (Table S2). Furthermore, NMDS analysis showed that treatments on Day 7 were clearly separated from the original (Fig. 3). On Day 7, the community distances to the original based on the Bray-Curtis distance were significantly higher in the KWP treatment than the BCA and CK treatments (LSD test, *P* < 0.05). However, the distances of the BCA and KWP treatments on Day 21 were close to the original compared to the CK (LSD test, *P* < 0.05), especially the BCA. The results indicated that the endophytic microbe community structure changed with time, and different applications of agents influenced the shifts among treatments.

## Correlation among endophytic microbial community and tobacco health

**Fig. 4:**
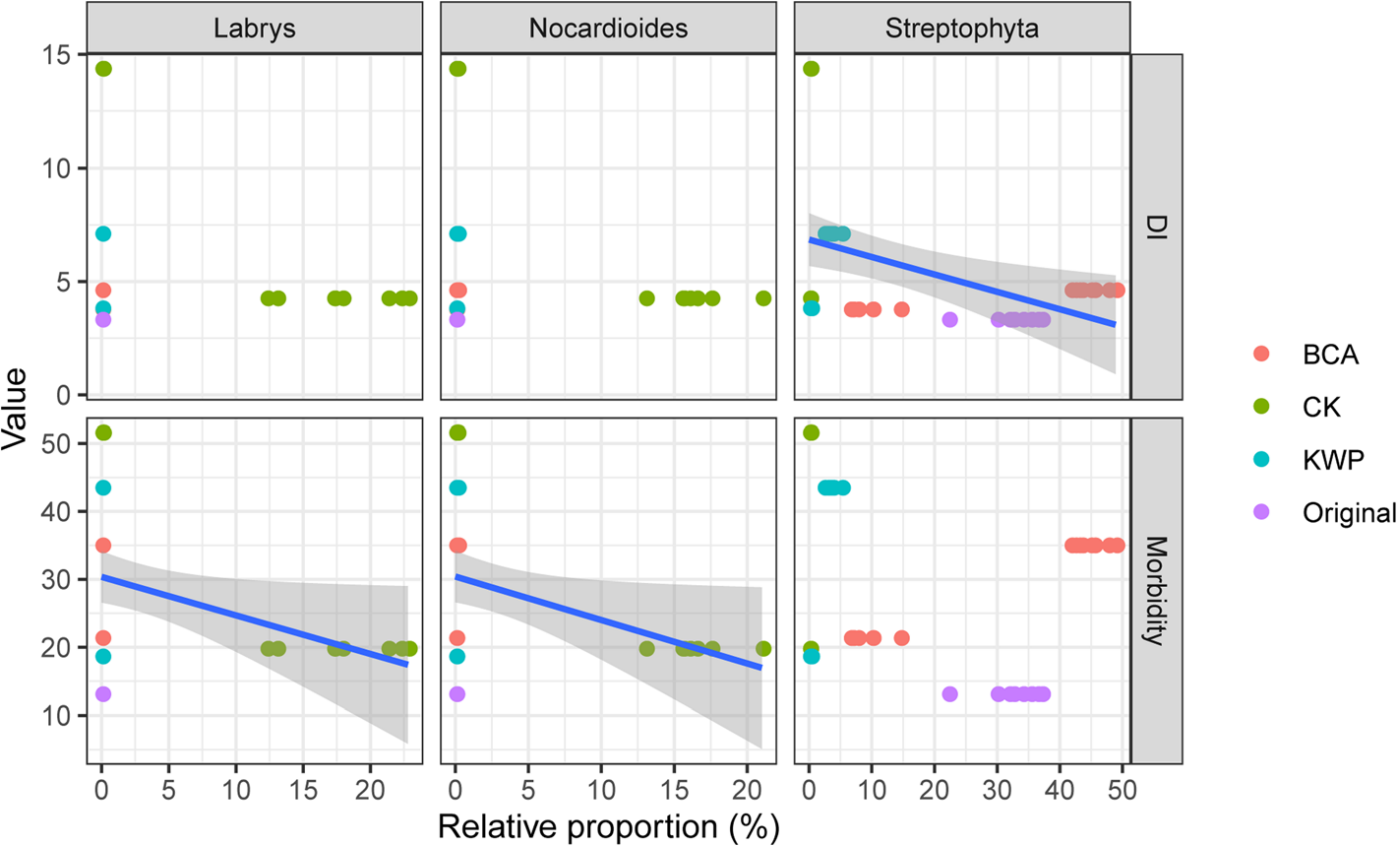
Correlation between plant health and three key genera (*Labrys*, *Nocardioides* and *Streptophyta*). DI disease index. The blue lines and shading represent the regression lines with 95% confidence intervals. Pearson correlation indexes and *p*-values are shown in Supplementary materials: Table S1

Correlation analyses were conducted between the microbial community (256 genera) and the disease index of wildfire disease, and 96 genera were significantly (*P* < 0.05) associated with the disease index (DI) or morbidity (Table S2). The correlations with most genera were positive, such as the abundant genera *Sphingomonas, Pseudomonas, Methylobacterium, Ochrobactrum, Tatumella, Acidovorax, Kosakonia, Bradyrhizobium, Nocardioides* and *Phenylobacterium*. Only three genera showed significant (*P* < 0.05) negative associations with the DI or morbidity (Fig. 4). The relative abundances of *Labrys* and *Nocardioides* were significantly and negatively correlated with morbidity (correlation = − 0.273, *p* = 0.042; correlation = − 0.276, *p* = 0.039). The relative abundance of *Streptophyta* was significantly and negatively correlated with the DI (correlation = − 0.358, *p* = 0.007). The results indicated that *Labrys, Nocardioides* and *Streptophyta* might play important roles in inhibiting wildfire disease. However, no OTUs for *Labrys* and few OTUs for *Nocardioides* were detected in the endophytic communities of either treatment, which demonstrated that members of *Labrys* and *Nocardioides* were not key species in suppressing wildfire disease of tobacco under these treatments.

### Endophytic community assembly of tobacco after BCA and KWP application

**Fig. 5:**
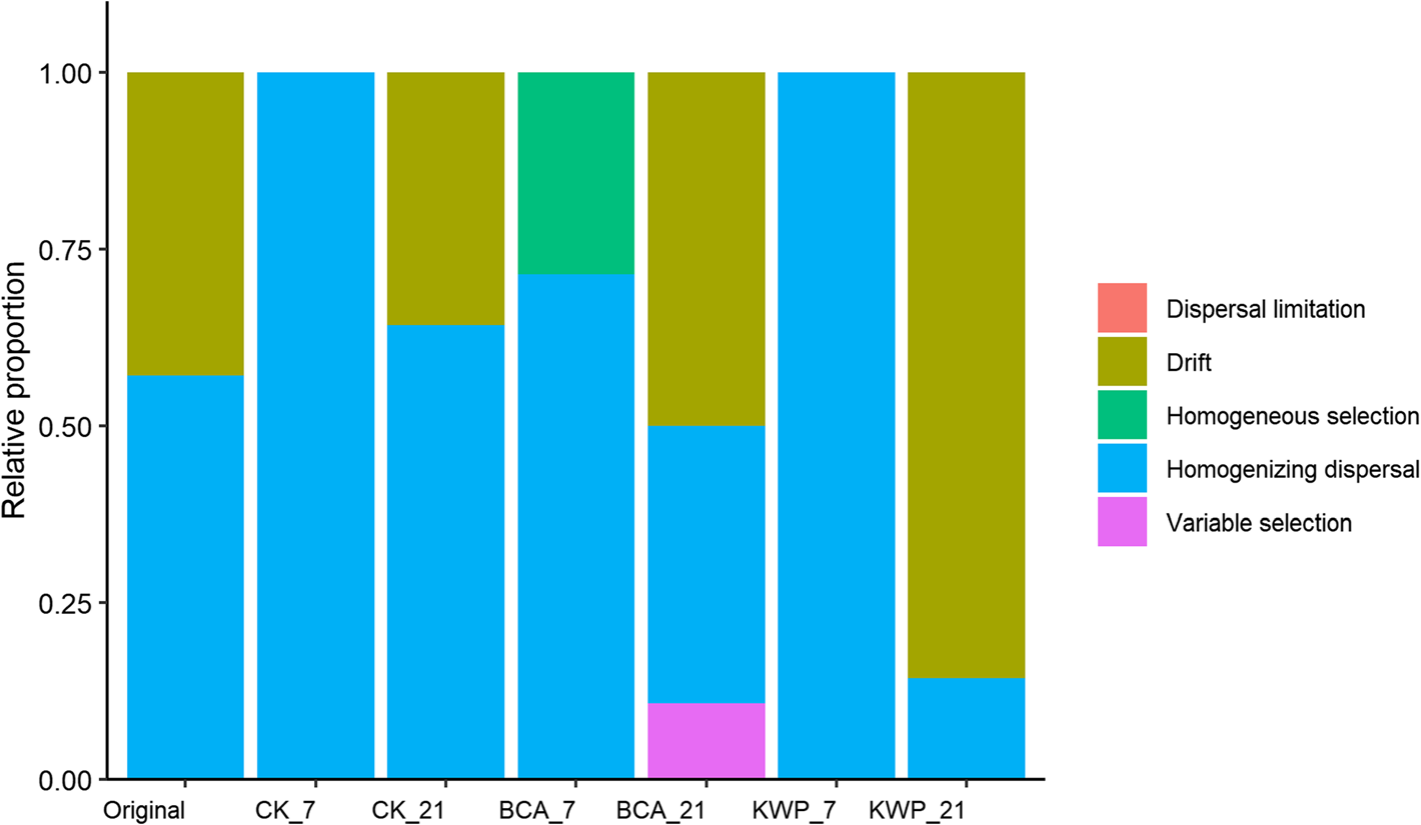
Relative proportions of the ecological processes in community assembly

To test the possible effects of agents on the original endophytic community assembly process, the relative contributions of major ecological processes (drift, homogeneous selection, homogenizing dispersal, and variable selection) were quantified. Figure 5 shows that processes regulating community turnover differed considerably between treatments and over time. Drift (43%) and dispersal (57%) were much more pronounced in the original samples (Day 0). The assembly processes of the endophytic microbial communities in the CK and KWP treatments were similar. The dominant process on Day 7 was dispersal (100%) and changed to drift and dispersal (total, 100%) by Day 21. Additionally, drift (86%) played a much more significant role than dispersal in KWP_21, while dispersal (64%) was more important in CK_21. In the BCA, the process was significantly different from that in the original, CK, and KWP treatments. Homogeneous selection and variable dispersal were the primary ecological processes in endophytic community assembly in the BCA samples. On Day 7, homogeneous selection (29%) and dispersal (71%) were the main processes after applying of the biological agent. On Day 21, the contribution of dispersal decreased (39%), and drift (50%) and variable selection (11%) replaced the role of homogeneous selection.

## Discussion

Plant disease is a serious threat to agricultural crops and is determined by a number of factors, including the plant growth states, pathogen virulence, endo- and epiphytic microbial communities, and external climate conditions [31, 32]. Recently, the use of plant-growth promoting bacteria (PGPB) based BCAs to suppress disease and improve yields has been utilized as an integrated crop management strategy [33, 34]. Previous research has outlined the responses of phyllosphere microbiota and tobacco disease resistance to two different biological control agents [14]. In the current study, the effects of a chemical agent (KWP) and a BCA on wildfire disease development in tobacco were compared. Both KWP and BCA showed control capacities for tobacco wildfire disease, with the control efficiency of the BCA reaching 67.99% being significantly higher than that of KWP (Tables 1 and S1). The endophytic microbial community responded differently to these two control methods (Figs. 2 and 3), which might suggest different control mechanisms. Furthermore, the BCA strengthened homogeneous and variable selection in community assembly, while KWP enhanced ecological drift (Fig. 4).

Exogenously applied agents might affect plants by activating their endophyte community [35], i.e., altering the population size or community structure of resident endophytes. In the present study, changes were observed in the community structure of tobacco leaf endophytic bacteria after spraying with the BCA and KWP treatments. On the one hand, the BCA and KWP application decreased the taxonomic diversity (Shannon index and Pielou evenness) of the leaf endophytic community (Fig. 2). The decreased species richness observed here was inconsistent with previous reports in which a more diverse microbial community in the soil or phyllosphere was found to be beneficial for tobacco disease suppression [14]. This might be attributed to the specific ecological niche of endophytic microorganisms. In general, innate endophytic bacteria are present in a latent state. When biotic or abiotic environmental stressors, such as phytopathogens [36], biological control agents [17] or elevated CO_2_ [37], are encountered, the endophytic community can be stimulated and activated [38]. Exogenously applied agents stimulate only some endophytic bacterial populations, promoting their rapid growth [38], thus resulting in a relatively simple community. For example, the upward trend of the rarefaction curves for the BCA and KWP treatments was steeper than CK on Day 7 (Figure S1), which indicated that the dominant members in samples accounted for a high proportion and might mask the real diversity. On the other hand, the NMDS analysis showed that the structure of the endophytic community was separated over time and by treatment (Fig. 3A). Compared to the BCA, KWP appeared to show a more significant effect on the endophytic community structure (Fig. 3B). However, the endophytic community structure of the BCA was similar to that of the control group on Day 7 (Fig. 3), and similar to the original state on Day 21. The results suggested that the endophytic community stimulated by the BCA had the ability to return to the original state, in which the disease infection was much lower. The impacts of exogenous factors on microbial community have been reported to be mitigated by the native microbes [39], and the direction of community succession might be related to native community traits and the type of exogenous factors [40]. Here, the low diversity of the endophytic community allows it to easily return to its original state. Moreover, the BCA, which was originally isolated from tobacco leaves, had high consistency and low risk to native tobacco microorganisms. Therefore, the changes and succession of the endophytic community under the BCA to the original state might be a potential way to decrease the disease incidence.

The application of both chemical and biological agents altered the microbial composition and improved plant health. Twenty-one days after treatment, *Sphingomonas* dominated the community in the KWP treatment and *Streptophyta* became the dominant genus in the BCA treatment (Fig. 1). In our previous study, we proposed that *Sphingomonas* spp. presented disease suppressive ability through competition for substrates with leaf-pathogenic *Pseudomonas syringae* [17] and promoted plant growth through the production of growth-stimulating factors [41]. However, previous studies have not focused on the control mechanism(s) of the genus *Streptophyta*, which is part of the phylum *Cyanobacteria*. To date, a number of *Cyanobacteria* species capable of producing a variety of biologically active compounds that can inhibit some bacteria and viruses have been identified [37, 42]. Furthermore, our results showed that the genus *Streptophyta* was significantly and negatively correlated with the disease index (Fig. 4 and Table S2), which implied that the *Streptophyta* genus is likely to antagonize wildfire disease in tobacco. Many studies have demonstrated that the antibacterial properties of *Cyanobacteria* members could be applied in controlling plant disease, such as chili pepper [43], tobacco [44], and tomato [45]. Similar to other members of the *Cyanobacteria* (*Nostoc* and *Microchaeta*), we speculate that *Streptophyta* may also have the potential to decrease the occurrence of tobacco wildfire disease. The variation in dominant species between the BCA and KWP suggested their different control mechanisms.

Biological control agents may alter the ecological process in microbial community assemblages by strengthening control effects. For instance, when put into a new environment, BCA bacteria compete with resident endophytes for resources, such as nutrition and living space [46], thus resulting in the decrease or extinction of less competitive species. This activity might provide a reasonable interpretation for the increase in homogenous selection in BCA treatment on Day 7. In this case, tobacco pathogens that have no competitive advantages would gradually dwindle, resulting in a temporary decrease of the disease index. However, the resource and niche availability levels in tobacco changed during plant growth. Consequently, the balance of the confrontation between biological control agents and tobacco pathogens based on competitiveness was broken, and some pathogens became more competitive than BCA bacteria, resulting in a secondary deterioration. At that point, the antibiotics secreted by BCA bacteria may play more important roles in defending against pathogens [47] and result in the ecological processes of variable selection during the late stage. This process effectively reduced the morbidity and disease index to a greater extent than chemical pesticides. Unlike biological control agents, the control effects of chemical pesticides were initially effective, but became less so with increasing use time. The nonselective antiseptic qualities of chemical pesticides eliminate native bacteria without resistance, including pathogenic bacteria. Such qualities lower compositional turnover and promote homogenizing dispersal as the initial ecological process in community assembly. During the later stage, the coexistence of ecologically similar species under the selection of chemical pesticides was mainly counteracted by ecological drift. However, antagonists to pathogenic bacteria also could dwindle as time passes. Thus, the ecological mechanisms regarding to the control effects of BCAs are different from those of chemical pesticides, and the ecological processes of variable selection could be a consideration for the efficacy of BCAs.

## Conclusions

This study demonstrates the different mechanisms of chemical and biological control agents on wildfire disease of tobacco. The control efficiency of the BCA reached 67.99%, which was significantly higher than that of KWP. Differences in disease control mechanisms by these two methods might be summarized based on three aspects. First, diversity in the BCA treatment was lower than in the control (CK) on Day 21, while that in the KWP treatment was significantly higher than that in the CK treatment. Second, the genus *Streptophyta* in BCA might play important roles in decreasing the occurrence of tobacco wildfire disease, while the major antagonistic bacteria in KWP might be *Sphingomonas*. Finally, the ecological processes in community assembly showed that the BCA strengthened homogeneous and variable selection in community assembly, while KWP enhanced ecological drift. These findings provide a scientific foundation for better understanding the control mechanisms of chemical and biological control methods from the view of microbial ecology.

## Methods

### Experimental design

A biological control agent and a chemical agent were used in a plot experiment. The chemical agent was a pesticide product against *Pseudomonas syringae* pv. *tabaci* containing kasugamycin wettable powder (4%). The experiment was conducted in Guiyang County, Hunan Province, China (112°64′17.3″, 5°80′81.2″) from May 2018 to July 2018. The experiment region was 180 m^2^, and was arranged as three treatments in a randomized complete block design with nine plots (three replicates for each treatment). The two treatment groups were sprayed with the BCA and KWP, and a control group was sprayed with sterile deionized water (CK) during the vigorous growth stage of tobacco when wildfire disease typically occurs and spreads rapidly. Tobacco plants (Yunyan87) were transplanted to experiment plots. Every block was 20 m^2^ and divided into three rows with a spacing of 1.1 m between rows. Fifteen tobacco plants were planted in each row with a spacing of 0.63 m between every two plants. Before the vigorous growth stage, plant health was monitored closely, and the agents were sprayed as soon as wildfire disease spots appeared in the plots. Agents were applied on plants once a week and sprayed four times in total, with the second application conducted 7 days after the first application, the third application performed after 14 days, and the fourth conducted after 21 days. Other agricultural management practices and fertilization regimes followed the local practice and were similar in all plots [48]. The experimental setup is shown in Figure S3.

The application of chemical agents was carried out according to the manufacturer’s instructions. Every plot required 1.8 g powder, and was sprayed onto tobacco leaves after being mixed with 6 L water. Both sides of tobacco leaves were sprayed carefully and equally with agent via a hand-held sprayer. The biological control agent used in this study was screened by our laboratory. We collected healthy leaves from healthy and infected tobacco plants in the field of Longshan County, and enriched microbial agents from exophytic and endophytic leaf microbial communities in 1/2 LB culture medium. Through antagonistic tests against *Pseudomonas syringae* pv. *Tabaci*, an agent from exophytic microbial communities, worked as a biological control agent and showed a great ability to inhibit the growth of pathogenic microorganisms in plate test. The biological control agent consisted of several genera, which were abundant (> 1%) (*Stenotrophomonas* (49.45%), *Achromobacter* (22.92%), *Enterobacter* (14.25%), *Ochrobactrum* (10.05%), and *Pseudomonas* (2.72%)), and rare (< 1%) (*Paenibacillus*, *Sphingomonas*, *Bacillus* and *Pseudochrobactrum*), as shown by 16S rRNA gene sequencing. The sequencing data of the agent have been made publicly available in the Sequence Read Archive (SRA) database of the NCBI under the following accession number PRJNA515831. After fermentation in 1/2 LB culture medium, the cell density of this agent reached 10^9^ cell/ml. The fermented agent was then diluted to a 1/4000 suspension with sterile deionized water. Every plot in the BCA treatment was sprayed with 6 L diluted suspension (containing 15 mL original fermentation). The spraying method was the same as that for the diluted chemical agent. Control plants in the blank treatment were treated with an equal amount of sterile deionized water.

### Tobacco disease incidence estimation and sample collection

Tobacco disease incidence was investigated in each plot with leaves as the base unit. In each plot, leaves of eight random plants in the middle row were investigated and the disease morbidity and disease index of wildfire disease were recorded [49], with three repetitions. Observations were performed before the first (Day 0), second (Day 7) and fourth (Day 21) applications of agents. The disease morbidity and disease index were calculated according to the following equations:

$$ \mathrm{Morbidity}\ \left(\%\right)=\left(\raisebox{1ex}{${n}^i$}\!\left/ \!\raisebox{-1ex}{$n$}\right.\right)\times 100 $$

$$ \mathrm{Disease}\ \mathrm{index}\ \left(\%\right)=\left[\sum \left(r\times {n}^i\right)/\left({n}^t\times R\right)\right]\times 100 $$

where *r* is the degree of disease infection, *n*^*i*^ is the number of infected plants, *n*^*t*^ is the number of tested tobacco plants, *R* is the value of the highest degree of disease infection, and *n* is the total number of plants in each plot. The degree of disease infection was classified according to six grades as previously reported including grade 0, grade 1, grade 3, grade 5, grade 7 and grade 9 [14].

To analyze the endophytic microbial communities, we collected leaf samples at Day 0 (original treatment), Day 7 (CK_7, BCA_7 and KWP_7), and Day 21 (CK_21, BCA_21 and KWP_21) when the tobacco disease incidence was estimated. Eight leaf samples were collected randomly from the fifteen investigated plants in each block. In the laboratory, leaf samples were shaken in phosphate-buffered saline (PBS) buffer to remove microbes from the leaf surfaces [14, 50]. The treated leaves were then stored at − 20 °C until DNA extraction.

### DNA extraction, PCR amplification, sequencing and data preprocessing

DNA extraction, PCR amplification and sequencing were performed as previously described [48, 51]. Briefly, microbial genomic DNA from tobacco endophytes was extracted using an EasyPure® Plant Genomic DNA Kit (TransGen Biotech, China), and the V4 region of the 16S rRNA gene was amplified with the primer pair 799F (5′-AACMGGATTAGATACCCKG-3′) and 1115R (5′-AGGGTTGCGC TCGTTG-3′) [52]. After purification using an OMEGA Gel Extraction Kit (Omega Bio-Tek, USA), amplification products were used for library construction and sequenced on Illumina MiSeq platform (Illumina, San Diego, CA, USA). Sequences were processed on a Galaxy pipeline (http://zhoulab5.rccc.ou.edu/) as previously described [53]. After quality trimming, low quality reads with QC scores < 20 and less than 200 bp long were removed [54]. Then Flash [55] was used to combine the pair-end reads with 20 to 250 bp overlap and lower than 5% mismatches. The combined sequences were assessed to remove short sequences, sequences containing N, and chimeras. Operational taxonomic units (OTUs) were carried out at 97% similarity level by UPARSE [56]. Finally, taxonomic assignment of representative sequences from each OTU was performed through the RDP [57] Classifier, with a minimal 50% confidence estimate.

### Data analysis

All the statistical analyses in the study were performed on the R statistical platform (version 3.6.1) [49, 58]. Multiple comparisons based on the least significant difference (LSD) test were performed to measure the difference in the tobacco wildfire disease index and community composition among treatments using the *agricolae* package. Different letters indicated significant differences (α = 0.05) among different treatments. Community diversity indices including Shannon-Weiner’s index (*H*) and Pielou evenness (*J*), were calculated with the ‘vegan V2.5–6’ package, and a multiple comparison was performed by the LSD test (*P* < 0.05) to compare the diversity index of different treatments. Different letters in the figure indicated significant differences. The community structure of endophytic microbes was measured by non-metric multidimensional scaling (NMDS), which was performed in R based on the Bray-Curtis distance matrix. Permutational analysis of variance (PERMANOVA) using Bray-Curtis distances was performed to reveal significant differences (*P* < 0.05) in the community compositions. Pearson correlations among genera and the disease index (DI) and morbidity (Mor) was performed, and a *P* value of less than 0.05 was considered statistically significant.

### Assembly process analysis

High-quality alignments were used to construct a maximum-likelihood tree using FastTree for further phylogenetic analysis. A previous research has confirmed that significant phylogenetic signals can be extended across relatively short distances by Mantel correlation (*P* < 0.05) [25]. Therefore, the βNTI and *RC*_*bray*_ (βNTI in combinations of the Bray-Curtis-based Raup-Crick) were further calculated to quantify the contribution of major ecological processes [59]. If βNTI < − 2 and βNTI > + 2, community turnover was determined by homogeneous and variable selection, respectively. Homogeneous selection caused similar community compositions because of a consistent selective environment among local scales, whereas variable selection caused changes in the community because of differences in the selective environments among local scales. If |βNTI| < 2 but *RC*_*bray*_ > + 0.95 or < − 0.95, then community turnover is governed by dispersal limitation or homogenizing dispersal processes, respectively. Dispersal limitation causes divergence in communities because of limited exchange among species, whereas homogenizing dispersal causes similar communities among local scales because of dispersal. But, if |βNTI| < 2 and |*RC*_*bray*_| < 0.95, then drift drives compositional turnover processes and is used to estimate the fraction (not dispersal or selection) that causes community differences. All analyses were run using the ‘picante V1.8’ and ‘ieggr V2.1’ packages in R.

## Supplementary Information

**Additional file 1.**

**Additional file 2.**

## Data Availability

All the 16S rRNA gene sequences were submitted to the NCBI database and the project number was PRJNA602108 and PRJNA515831.

## Abbreviations

BCA: Biological control agent
KWP: Kasugamycin wettable powder
CK: Control group
LSD: Least significant difference
H: Shannon-Weiner’s index
J: Pielou evenness
DI: Disease index
Mor: Morbidity
PGPB: Plant-growth promoting bacteria
LB: Luria-Bertani
SRA: Sequence Read Archive
NCBI: National Center for Biotechnology Information
PBS: Phosphate-buffered saline
NMDS: Non-metric multidimensional scaling
PERMANOVA/ADONIS: Permutational analysis of variance
NTI: Nearest taxon index
