# Supplementary material for: Ecological strategies of biological and chemical control agents on wildfire disease of tobacco (Nicotiana tabacum L.)
Source: BMC Microbiol. 2021 Jun 17;21:184. doi: 10.1186/s12866-021-02237-8 (PMC8212473; doi:10.1186/s12866-021-02237-8)
Supplement: Supplementary file 1 — Additional file 1. [file 12866_2021_2237_MOESM1_ESM.pdf]

BMC Microbiology

Supplementary materials

Ecological strategies of biological and chemical control agents on wildfire disease of tobacco (*Nicotiana tabacum* L.)

Tianbo Liu[1,3†], Yabing Gu[2†], Zhicheng Zhou[3], Zhenghua Liu[2], Huaqun Yin[2], Chong Qin[2], Tuyong Yi[1]*, Jiemeng Tao[4]*

[1] College of Plant Protection, Hunan Agricultural University, Changsha, China;

[2] School of Minerals Processing and Bioengineering, Central South University, Changsha, China;

[3] Central South Agricultural Experiment Station of China Tobacco, Changsha, China;

[4] China Tobacco Gene Research Center, Zhengzhou Tobacco Research Institute of CNTC, Zhengzhou, China

† Tianbo Liu and Yabing Gu contributed equally to this work.

* Address Correspondence to Jiemeng Tao and Tuyong Yi, E-mail: taojiemeng@csu.edu.cn; yituyong@hunau.net

Tel: +86(731)88830546

Fax: +86(731)88830546

SM: Figures

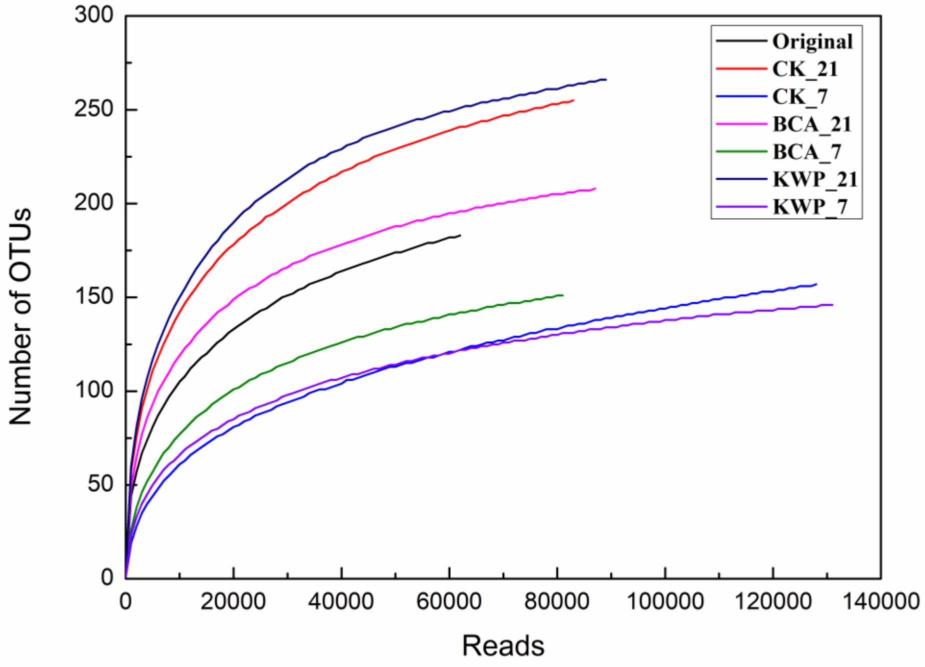

Figure S1. Rarefaction curve of 16S rRNA gene sequencing in each treatment, including control group (CK) and treatments of biological agent (BCA) and chemical control agent (KWP). The number in front of "_" is the number of experimental days (0 day for CK group labeled as "Original"), while the number behind "_" is the serial number of replicates.

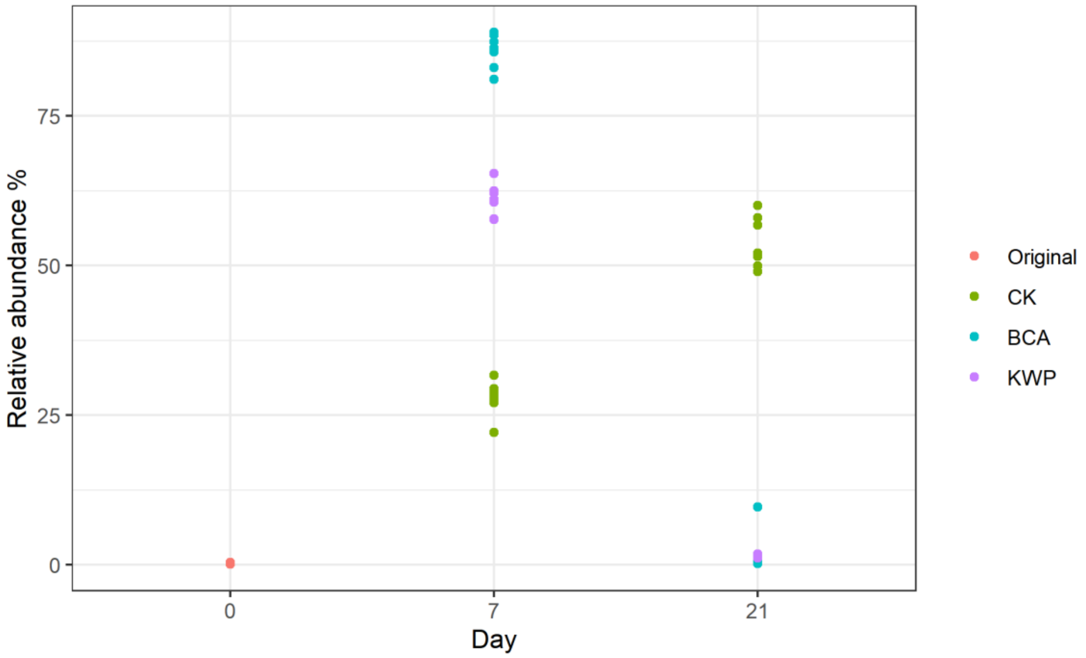

Figure S2. Relative abundance of *Pseudomonas* genus across the groups of control groups (CK), biological control agent (BCA) and chemical control agent (KWP).

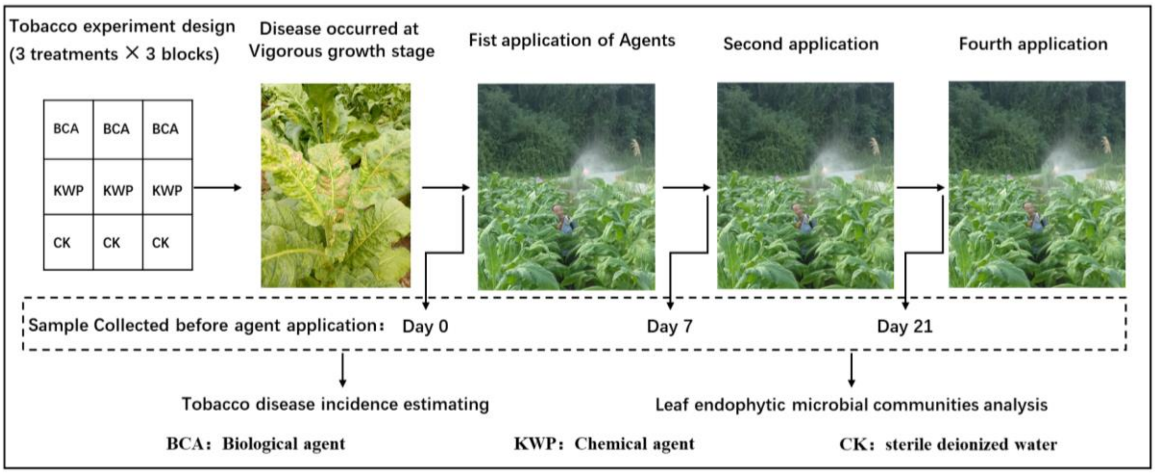

Figure S3. Schematic view of the field experiment. The whole experiment consisted of three treatments (BCA, KWP and CK). At vigorous growth stage when the wildfire disease occurs and spread widely, agents were sprayed in tobacco field. After seven days, the second applied of agents were conducted. The third application was performed after 14 days, and the fourth was conducted after 21 days. Then tobacco disease incidence was estimated and leaf samples were collected before the first (Day 0), second (Day 7) and fourth (Day 21) applications.

Table S1 Morbidity of tobacco wildfire disease under different treatments

Treatment	0 day	7 day	21 day
BCA	13.25±0.90a	21.26±3.24a	34.91±2.26c
KWP	13.00±0.67a	18.55±0.12a	43.4±2.03b
CK	13.31±0.55a	19.69±1.17a	51.48±4.01a

Different letters indicate significant difference at 0.05 by multiple comparisons based on the means of least significant difference and a grouping of treatments.

Table S2 Significant correlations between the relative abundance of 256 genera and the incidence indices of disease index (DI) and morbidity (Mor).

Genus	DI		Mor	
	Correlation	*p*	Correlation	*p*
Achromobacter	0.380087	0.003859	0.287305	0.031794
Aciditerrimonas	0.313405	0.018672	0.22701	0.092468
Acidovorax	0.947597	1.97E-28	0.691262	3.69E-09
Acinetobacter	0.63246	1.70E-07	0.656224	4.00E-08
Aminobacter	0.650408	5.78E-08	0.468505	0.000271
Amnibacterium	0.302611	0.023397	0.270212	0.043998
Anaerococcus	0.313405	0.018672	0.22701	0.092468
Aquabacterium	0.354196	0.007402	0.493589	0.000111
Armatimonadetes_gp5	0.313405	0.018672	0.22701	0.092468
Aureimonas	0.4435	0.000618	0.304636	0.022441
Bdellovibrio	0.294182	0.027753	0.44688	0.000555
Blastococcus	0.33025	0.012926	0.348869	0.008409
Bosea	0.59143	1.59E-06	0.451694	0.000475
Bradyrhizobium	0.938688	1.22E-26	0.681562	7.37E-09
Brevibacterium	0.437298	0.000752	0.562549	6.41E-06
Brevundimonas	0.098736	0.469076	0.467137	0.000284
Burkholderia	0.351883	0.007826	0.357673	0.006802
Capnocytophaga	0.313405	0.018672	0.22701	0.092468
Chelativorans	0.516924	4.52E-05	0.404832	0.001969
Chelatococcus	0.510685	5.79E-05	0.369906	0.005017
Chitinophaga	0.526048	3.13E-05	0.432208	0.00088
Chryseobacterium	0.334597	0.011718	0.327087	0.013871
Citreicella	0.27337	0.041492	0.187232	0.167043
Corynebacterium	0.264151	0.049156	0.31073	0.01976
Curtobacterium	0.391391	0.002856	0.299397	0.024984
Delftia	-0.00903	0.947363	0.266159	0.047395
Desulfobacca	0.313405	0.018672	0.22701	0.092468

Dietzia	0.342002	0.009883	0.362229	0.006081
Escherichia/Shigella	0.033839	0.804453	0.3111	0.019607
Fluviicola	0.302611	0.023397	0.270212	0.043998
Hyphomicrobium	0.53444	2.21E-05	0.394794	0.002603
Hyphomonas	0.447307	0.000548	0.324	0.01485
Janibacter	0.406954	0.001854	0.311821	0.01931
Jatrophihabitans	0.346335	0.008929	0.264181	0.049129
Kocuria	0.519724	4.04E-05	0.376454	0.004242
Kosakonia	0.120263	0.377302	0.421496	0.001215
Kytococcus	0.255701	0.05716	0.266034	0.047503
Labrys	-0.17821	0.188832	-0.27317	0.041643
Leucobacter	0.313405	0.018672	0.22701	0.092468
Lysinimicrobium	0.313405	0.018672	0.22701	0.092468
Lysobacter	0.509904	5.96E-05	0.369341	0.005089
Marmoricola	0.304764	0.022381	0.349172	0.008349
Mesorhizobium	0.668849	1.76E-08	0.491251	0.000121
Methylobacterium	0.937427	2.08E-26	0.670155	1.61E-08
Methyloversatilis	0.290568	0.029819	0.224287	0.096553
Microbacterium	0.481499	0.000172	0.413764	0.001525
Mycobacterium	0.169296	0.21227	0.316274	0.017563
Neochlamydia	0.237152	0.078432	0.304996	0.022274
Neorhizobium	0.338432	0.010734	0.244488	0.069373
Nevskia	0.156466	0.249493	0.326067	0.014188
Nocardioides	-0.1806	0.182861	-0.27622	0.039332
Ochrobactrum	0.199523	0.140404	0.48229	0.000167
Paenibacillus	0.447307	0.000548	0.321813	0.015579
Paenisporosarcina	0.313405	0.018672	0.22701	0.092468
Parafilimonas	0.395067	0.002584	0.35277	0.007661
Parasegetibacter	0.703489	1.48E-09	0.568092	4.95E-06
Pedobacter	0.300945	0.024209	0.198206	0.143096
Pedomicrobium	0.313405	0.018672	0.22701	0.092468
Pelomonas	0.176309	0.193659	0.38976	0.002985
Peptococcus	0.313405	0.018672	0.22701	0.092468
Peredibacter	0.447307	0.000548	0.324	0.01485
Phenylobacterium	0.946173	4.00E-28	0.696591	2.49E-09
Phreatobacter	0.201413	0.136608	0.300347	0.024506
Phyllobacterium	0.590532	1.66E-06	0.428971	0.000971
Pigmentiphaga	0.301297	0.024035	0.25373	0.059171
Porphyrobacter	0.316928	0.017318	0.367999	0.005264
Prosthecomicrobium	0.313405	0.018672	0.22701	0.092468
Proteus	0.313405	0.018672	0.22701	0.092468
Pseudolabrys	0.528112	2.88E-05	0.369759	0.005035
Psychrobacter	0.446944	0.000554	0.351558	0.007887
Ralstonia	0.528813	2.79E-05	0.483311	0.000161

Reyranella	0.560988	6.88E-06	0.42978	0.000947
Rheinheimera	0.276788	0.038913	0.220994	0.101677
Rhizobium	0.511998	5.50E-05	0.42137	0.00122
Rhodoblastus	0.313405	0.018672	0.22701	0.092468
Rhodopseudomonas	0.640466	1.06E-07	0.603722	8.41E-07
Rickettsia	0.25892	0.053995	0.273719	0.041222
Rubellimicrobium	0.313405	0.018672	0.22701	0.092468
Rubrobacter	0.313405	0.018672	0.22701	0.092468
Saccharibacteria_genera_incertae_sedis	0.244719	0.069102	0.324922	0.014552
Sanguibacter	0.313405	0.018672	0.22701	0.092468
Sediminibacterium	0.920345	1.13E-23	0.666637	2.04E-08
Shewanella	0.102201	0.453545	0.271532	0.042936
Solimonas	0.242206	0.072097	0.434541	0.000819
Sphingobium	0.394498	0.002624	0.54393	1.47E-05
Sphingopyxis	0.493972	0.000109	0.42624	0.001055
Streptomyces	0.153693	0.258087	0.290508	0.029855
Streptophyta	-0.35842	0.006678	-0.16695	0.218773
Taonella	0.587883	1.90E-06	0.471378	0.000245
Tardiphaga	0.628144	2.19E-07	0.458015	0.000386
Thermus	0.498275	9.31E-05	0.612943	5.13E-07
Unclassified	-0.42209	0.001194	-0.28402	0.033888
Varibaculum	0.313405	0.018672	0.22701	0.092468
Williamsia	0.425331	0.001084	0.333101	0.012122
Xanthobacter	0.39861	0.002344	0.288727	0.03092
Yimella	0.313405	0.018672	0.22701	0.092468
